# Analysis of physical and mechanical behaviors and microscopic mineral characteristics of thermally damaged granite

**DOI:** 10.1038/s41598-024-65752-4

**Published:** 2024-06-26

**Authors:** Lei Peng, Xianglong Li, Xin Peng, Yunchuan Gan, Jianguo Wang

**Affiliations:** 1 Faculty of Land Resources Engineering, Kunming University of Science and Technology, Kunming, 650093 Yunnan China; 2 Advanced Blasting Technology Engineering Research Center of Yunnan Provincial Department of Education, Kunming, 650093 Yunnan China

**Keywords:** Thermal damage, Granite, Physical and mechanical behavior, Mineral composition, Microscopic characteristics, Natural hazards, Civil engineering

## Abstract

Temperature’s influence on the physical and mechanical properties of rocks is a crucial concern for the rational design of deep rock engineering structures and the assurance of their long-term stability. To systematically comprehend the impact of the evolution of mineral composition and micro characteristics on the physical and mechanical behavior of thermally damaged granite, we observed the microscopic structural defects inside the rocks with a polarizing microscope and revealed the thermal damage mechanism of granite from a microscopic perspective by combining ultrasound detection and XRD phase characteristic analysis. The results show that the physical properties of the specimens changed significantly at three characteristic temperature points: 400 °C, 800 °C, and 1000 °C. Under high temperature conditions, the diffraction intensity of all minerals in granite, except for quartz, generally decreased, and stable minerals decomposed. Albite and potash feldspar decomposed to form anorthoclase, thereby reducing the structural stability of the rock material. In addition, the peak width of various minerals decreased to varying degrees with increasing temperature. The increase in mineral volume further damaged the internal structure of the rock material while promoting the transformation from grain boundary to intergranular cracks and from intragranular cracks to transgranular cracks, ultimately forming a interconnected crack network. Thermal damage significantly reduced the longitudinal wave velocity, uniaxial compressive strength, and elastic modulus of the specimens, while the stress–strain curve relationship indicated that the specimens underwent two opposite processes of transformation from brittleness to ductility and then from ductility to brittleness. The thermal damage threshold of granite in this study was 600 °C.

## Introduction

As various types of underground engineering projects continue to develop deeper, the geothermal gradient increases, leading to significant thermodynamic effects on deep rock masses. These effects have triggered secondary disasters in many large geotechnical engineering structures, such as deep mining, deep tunnels, oil or gas drilling, geothermal resource development, and radioactive waste disposal^1–6^. Granite, being one of the most widely used materials in construction and civil engineering, has its physical and mechanical properties form the foundation for the stability of deep underground space structures. After exposure to high temperatures, the micro and macro characteristics of rock materials undergo significant changes, causing irreversible damage to the crystal structure and cementation within the rock, thereby altering its physical and mechanical properties. Therefore, conducting experimental research on the mechanical properties of thermally damaged rocks is of great significance for the rational design of deep rock engineering structures and maintaining their long-term stability.

In terms of physical properties, most research focuses on parameters such as mass, volume, density, porosity, permeability, and thermal conductivity^7–11^. The longitudinal wave velocity is highly sensitive to the discontinuous microstructures present in rock specimens, which can be used to detect the behavior of microcrack propagation. There is often a threshold temperature at which the wave velocity significantly decreases^12,13^. Fan et al.^14^ studied the propagation characteristics of longitudinal waves in granite after different high-temperature treatments using a pendulum impact device and successfully introduced the propagation coefficient (wave velocity attenuation coefficient) to describe the attenuation law of longitudinal waves in thermally damaged granite. Jiang et al.^15^ established a velocity-permeability model based on porosity as the medium, which allows the permeability of rocks at any temperature to be obtained using the wave velocity.

Deep rock formations are often subject to high-temperature effects and have microstructural defects. Due to the complexity of mineral compositions within the rock and different thermal expansion coefficients of various minerals, structural thermal stress occurs between minerals at high temperatures. This leads to the development and propagation of thermal-induced microcracks within the rock, ultimately weakening its strength and stiffness, thereby affecting the quality and durability of deep rock formations^16^. Various types of thermal-induced microcracks include intracrystalline cracks, grain boundary cracks, and transgranular cracks^8,12,17–20^. Niu et al.^21–23^ employed high-speed camera, microseismic monitoring and borehole imaging techniques to observe the fracturing process of rocks. In terms of mineral studies, due to high-temperature effects, feldspar undergoes continuous alteration and decomposes into clay minerals such as kaolinite and illite^24,25^, while the crystal structure properties of mica also undergo significant changes^26,27^. However, in granite, feldspar, biotite, and quartz minerals experience creep at high temperatures, resulting in a significant decrease in elastic modulus and a notable reduction in the material’s resistance to deformation^28^. He et al.^29^ found that the deformation of different mineral particles caused by heating is non-uniform. During the heating process, feldspar expands by 5.29–7.62%, while quartz and mica are compressed by 3.74–6.27% and 3.65–6.16% respectively. Expansion deformation gradually recovers during constant temperature and cooling processes, but irreversible deformation still exists.

In terms of mechanical properties, the fracture toughness of granite decreases with increasing heat treatment temperature, often exhibiting a threshold temperature. Beyond this threshold temperature, the fracture toughness value significantly drops due to structural and chemical changes in the constituent minerals^26,30,31^. Qin et al.^12^ summarized the effects of temperature on parameters such as elastic modulus, axial strain, UCS, cohesion, and internal friction angle. Their research determined 600 °C as the threshold and transition temperature for the mechanical properties and failure modes of the tested granite. In recent years, many scholars have combined elastoplastic theory, fracture mechanics, and damage statistical theory to establish a series of rock constitutive models based on axial stress-axial strain response through rock mechanics tests, to describe the effects of high temperatures on rock mechanical behavior^32–35^. Kang et al.^36^ studied fine-grained, medium-grained, and coarse-grained granite subjected to different temperature treatments and found that after 800 °C heat treatment, the UCS of coarse-grained granite decreased to 22.38%, whereas the UCS of medium-grained and fine-grained granite decreased to 43.52% and 47.54%, respectively.

Although many experiments have been conducted to study the physical and mechanical behavior of high-temperature rocks, the mechanism of how high temperatures affect the physical and mechanical properties of rocks is still not fully understood. In addition, existing research mostly focuses on the physical and macroscopic mechanical properties of thermally damaged rocks, and there is limited research on the impact of analyzing the evolution of mineral composition and microscopic characteristics in rocks with temperature on their physical and mechanical properties. In this study, we investigated the physical and mechanical behavior of a coarse-grained granite at temperatures ranging from room temperature (25 °C) to 1200 °C. The variations of specimen mass, volume, density, longitudinal wave velocity, UCS value, elastic modulus, peak strain, stress–strain relation, and failure mode with temperature were analyzed. Subsequently, the thermal damage mechanism of the granite was revealed from a microscopic level through the examination of microstructure, mineral characteristics, chemical composition, molecular structure, diffraction peak intensity, and full width at half maximum (FWHM) of thermally damaged granite.

## Experimental design

### Description of rock specimens

**Figure 1:**
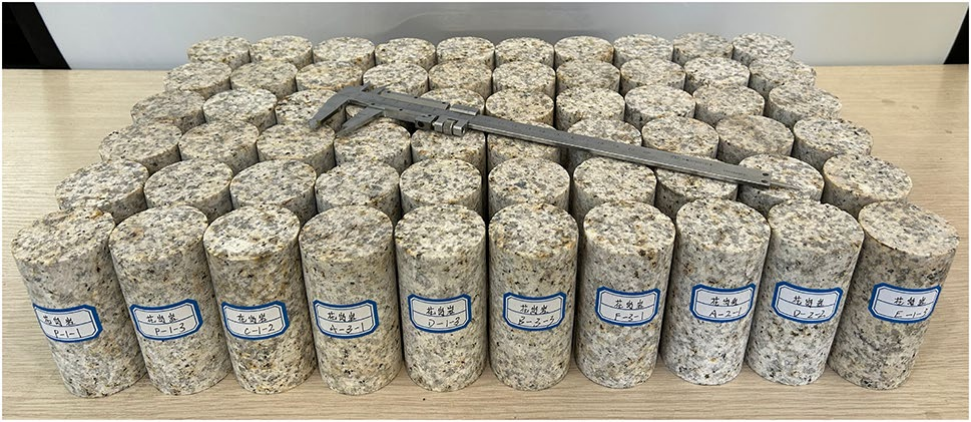
Granite specimen for testing.

The granite samples used in the experiment were collected from Wenshang County, Jining City, Shandong Province, China. To ensure the uniformity of the rock specimens used in the tests, large rock blocks with rough dimensions of 100 × 50 × 30 cm^3^ were obtained from the mine. Subsequently, 60 cylindrical specimens with a diameter of 50 mm and a length of 100 mm were extracted from the rock blocks using a wire cutting machine, as shown in Fig. 1. All rock specimens were carefully ground and polished at the ends, ensuring that their non-parallelism and non-perpendicularity were both less than 0.05 mm. The height and diameter error of the specimens did not exceed 0.3 mm. The maximum deviation of the end face from being perpendicular to the specimen axis was no more than 0.25°, meeting the specifications recommended by ISRM^37^.

The texture and composition of the rock are relatively uniform, with a grain size of approximately 2–3 mm. XRD tests revealed that the rock is primarily composed of quartz, potash feldspar, and plagioclase, with a small amount of biotite. The average UCS, volume, mass, density, and P-wave velocity of the rock are 113.54 MPa, 195.54 cm^3^, 505.5 g, 2.57 g/cm^3^, and 3067 m/s, respectively.

### Testing instrument

1. YAW-600 Microcomputer Controlled Hydraulic Servo Pressure Testing Machine.
2. XW7L-12 High Temperature Box-type Resistance Furnace, with a maximum heating temperature of 2000 °C.
3. X’Pert3 Powder Multifunctional Powder X-ray Diffractometer. X-ray diffraction using Cu target, with a wavelength λ = 1.54056 Å, detection range (2θ): 5°–90°, detection speed: 10°/min, step size 0.026°/s.
4. Axios-Max (PW4400/40) Wavelength Dispersive X-ray Fluorescence Spectrometer developed by PANalytical in the Netherlands. Power: 4 kW, test element range: F-U.
5. Nikon E600 Research-grade Polarizing Microscope.
6. RSM-SY6 (C) Non-metal Ultrasonic Testing Instrument (detecting probe frequency is 50 kHz).

### Heating procedure

The rapid heating rate often leads to uneven heating of the rock and rapid rupture, and even the phenomenon of debris shedding^38–40^. Tian et al.^41,42^ summarized the heating rate and constant temperature time selected by many scholars and found that setting the heating rate to 2–10 °C/min and the constant temperature time to 1–2 h had the best effect. The heating device used in the experiment is an XW7L-12 box type resistance furnace, which consists of a control box and a furnace chamber. The maximum working temperature is 1800 °C, with an allowable error of 1–3 °C. Heat each specimen to the predetermined temperature at a heating rate of 10 °C/min while ensuring uniform heating. After the preset temperature is reached, it is kept constant for 2 h to ensure that the granite specimens have sufficient thermal damage. Once the constant temperature is finished, the furnace door is opened and the specimens are taken out of the furnace and cooled slowly to room temperature under natural ventilation. To avoid reactions between the high-temperature treated rock specimens and moisture in the air, The Tesite WGLL-230BE electric drying oven is used to dry and store the high-temperature treated specimens for subsequent use in the experiment.

**Figure 2:**
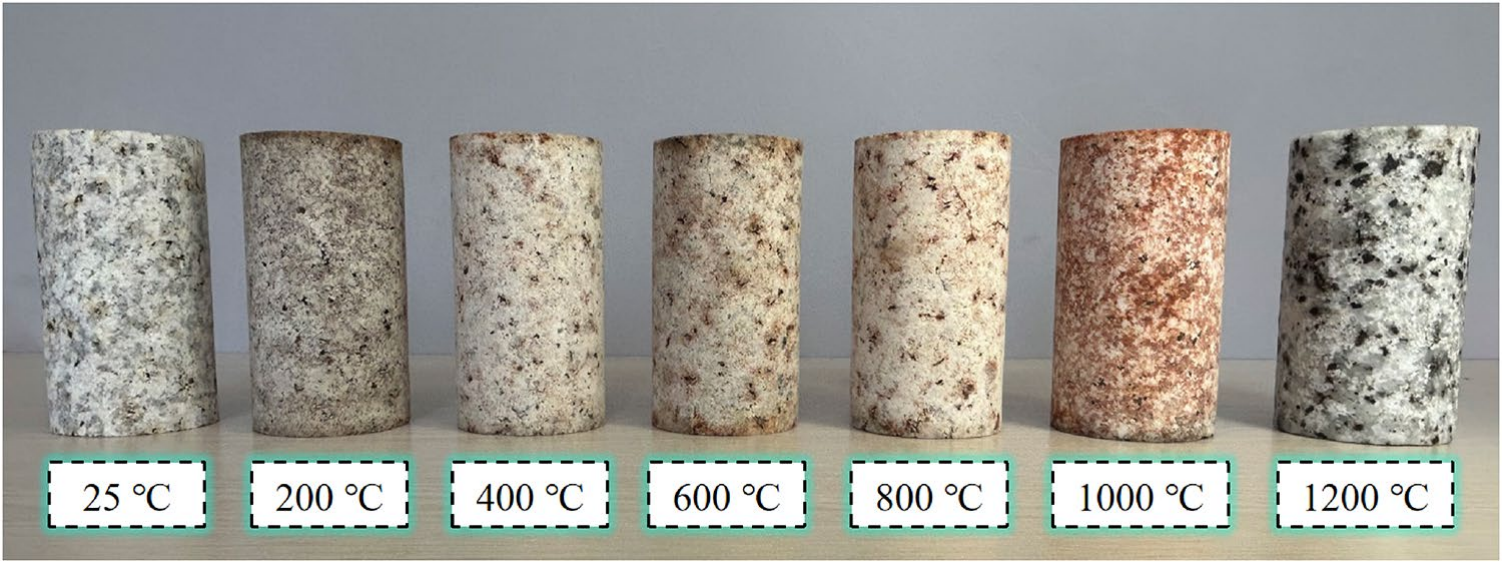
Granite specimens treated at different temperatures and naturally cooled.

When the granite specimen is heated to 1400 °C, the integrity of the specimen is destroyed due to complete melting. Therefore, the maximum heating temperature was limited to 1200 °C and divided into 7 groups, which are room temperature (25 °C), 200 °C, 400 °C, 600 °C, 800 °C, 1000 °C and 1200 °C. The number of specimens in each group was 3. The granite specimens after heating and natural cooling at each temperature gradient are shown in Fig. 2.

### Test procedures and steps

Before the uniaxial compression testing, the basic physical indicators of the granite specimens after different temperature treatments were measured, namely mass, size, and density (note: each indicator was measured three times and then averaged. When measuring size, different positions of rock specimens were selected). The longitudinal wave velocity of specimens was measured using non-metal sonic detector. During the measurement, use white Vaseline to eliminate air between the surface of the specimen and the transducers to ensure that energy is effectively transmitted to the transducers, and each specimen was detected three times.

**Figure 3:**
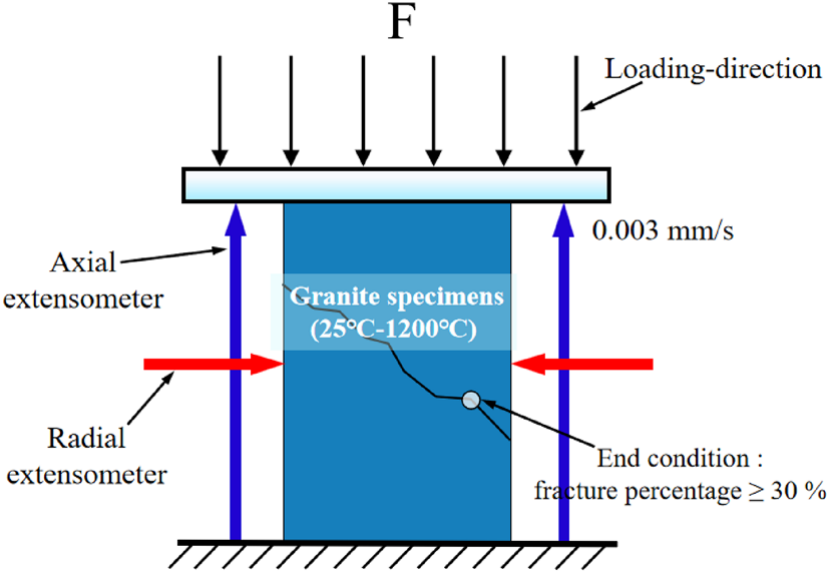
Uniaxial compression schematic diagram.

Uniaxial compression test were performed using a hydraulic servo-control testing system with a maximum load capacity of 300 kN. The measurement range of the axial and radial extensometers are 6 mm and 3 mm respectively. The extensometers were carefully calibrated before testing. The test was carried out using an axial displacement control loading with a rate of 0.003 mm/s. The axial strain, radial strain and axial stress during the loading process were recorded. The uniaxial compression loading process is shown in Fig. 3.

**Figure 4:**
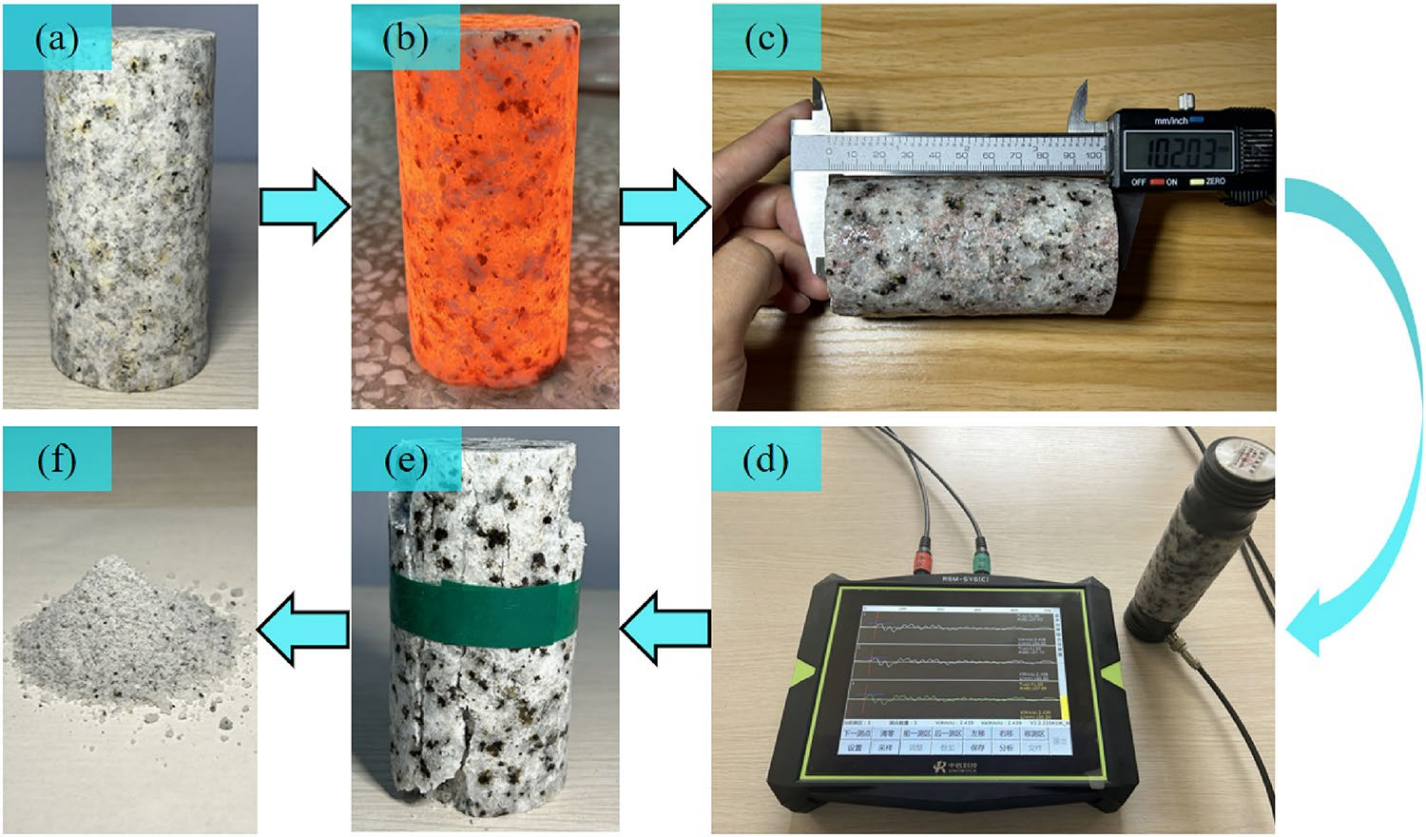
Test process: (**a**) rock specimen processing. (**b**) High temperature heating. (**c**) Determination of basic physical indicators. (**d**) Determination of longitudinal wave velocity. (**e**) Uniaxial compression experiment. (**f**) XRF and XRD analysis and determination.

After the uniaxial compression test is completed, the destroyed granite specimens are recovered, and rock specimens from the granite specimens after different temperature treatments are collected. The collected specimens are classified and then ground into powder specimens with a mass of 5 g and a uniform particle size (particle size of 320 mesh/40 μm) using natural agate pestles. The handheld powder specimens have a flour-like texture and no particle sense. These powder specimens are used for the determination and analysis of granite element content (XRF) and mineral composition (XRD). The complete test process is shown in Fig. 4.

## Experimental results

### Analysis of physical changes of granite specimen

To more intuitively demonstrate the trends of various basic physical indicators of granite specimens with temperature changes, the volume change fraction *η*_*V*_, mass change fraction *η*_m_, and density change fraction *η*_*ρ*_ were defined. Meanwhile, the volume growth rate *K*_*V*_, mass loss rate *K*_m_, and density loss rate *K*_*ρ*_ were used to characterize the changes in volume, mass, and density of granite after natural cooling from high temperatures. The volume growth rate is the percentage of the volume increase of the rock specimen after natural cooling from high temperature relative to the original specimen volume. The mass loss rate is the percentage of the mass decrease of the rock specimen after natural cooling from high temperature relative to the original specimen mass. The density loss rate is the percentage of the density decrease of the rock specimen after natural cooling from high temperature relative to the original specimen density. The physical test parameters of granite specimens before and after natural cooling from high temperatures are shown in Table 1. The relationships and calculation formulas for the change fractions and change rates of various physical indicators are shown in Eqs. (1)–(6).

1 $$\eta_{V} = \frac{{V_{2} }}{{V_{1} }} \times 100\%$$

2 $$K_{V} = \frac{{V_{2} - V_{1} }}{{V_{1} }} \times 100\% = \left( {\eta_{V} - 1} \right) \times 100\%$$

3 $$\eta_{m} = \frac{{m_{2} }}{{m_{1} }} \times 100\%$$

4 $$K_{m} = \frac{{m_{1} - m_{2} }}{{m_{1} }} \times 100\% = \left( {1 - \eta_{m} } \right) \times 100\%$$

5 $$\eta_{\rho } = \frac{{\rho_{1} }}{{\rho_{2} }} \times 100\%$$

6 $$K_{\rho } = \frac{{\rho_{1} - \rho_{2} }}{{\rho_{1} }} \times 100\% = \left( {1 - \eta_{\rho } } \right) \times 100\%$$

**Table 1:** Physical test parameters of granite specimens treated at different temperatures.

Number	*T* (℃)	Mass/(g)		Volume (cm^3^)		Density (g cm^-3^)		Wave velocity (m s^-1^)	
		*m*_1_ (before)	*m*_2_ (after)	*V*_1_ (before)	*V*_2_ (after)	*ρ*_1_ (before)	*ρ*_2_ (after)	*V*_P1_ (before)	*V*_P2_ (after)
P-1	25	501.5	501.5	195.77	195.77	2.5616	2.5616	3109	3109
P-2	25	502.4	502.4	195.53	195.53	2.5694	2.5694	3023	3023
P-3	25	503.6	503.6	195.32	195.32	2.5783	2.5783	3068	3068
A-1	200	502.8	500.7	195.58	195.82	2.5708	2.5569	3192	2381
A-2	200	502.4	500.7	195.49	195.68	2.5699	2.5587	3159	2382
A-3	200	505.6	503.9	196.09	195.70	2.5784	2.5748	2885	2143
B-1	400	503.3	502.3	195.62	196.49	2.5728	2.5563	2885	1685
B-2	400	499.6	498.6	194.86	196.53	2.5638	2.5370	2913	1639
B-3	400	503.6	502.4	195.15	196.60	2.5805	2.5554	3030	1754
C-1	600	498.8	497.6	194.08	199.96	2.5701	2.4884	3030	721
C-2	600	504.4	502.8	196.35	202.54	2.5688	2.4824	2941	699
C-3	600	503.8	502.1	195.64	200.24	2.5751	2.5074	3062	741
D-1	800	499.1	494.8	195.55	214.27	2.5522	2.3092	2985	450
D-2	800	502.3	500.8	195.37	204.27	2.5710	2.4516	3014	516
D-3	800	498.9	497.3	194.61	202.68	2.5635	2.4536	3085	513
E-1	1000	502	500.6	195.15	206.51	2.5723	2.4240	3159	399
E-2	1000	504.7	503	195.19	206.67	2.5856	2.4338	3372	407
E-3	1000	504.5	502.8	195.88	208.49	2.5755	2.4116	3226	389
F-1	1200	502.8	500.7	196.55	212.06	2.5581	2.3611	3104	911
F-2	1200	502.4	500.7	195.51	211.55	2.5696	2.3668	3041	977
F-3	1200	505.6	503.9	196.51	211.80	2.5728	2.3791	3113	977

Numbers P, A, B, C, D, E and F represent rock specimens at room temperature (25 °C), 200 °C, 400 °C, 600 °C, 800 °C, 1000 °C and 1200 °C respectively. *m*_1_,* V*_1_, *ρ*_1_ and *V*_*P*1_ are the mass, volume, density and longitudinal wave velocity of the rock specimen before heating, respectively. *m*_2_, *V*_2_, *ρ*_2_ and *V*_*P2*_ are the mass, volume, density and longitudinal wave velocity of the rock specimen after natural cooling at room temperature, respectively.

In the equation, *V*_1_, *m*_1_, and *ρ*_1_ represent the volume, mass, and density of the original rock specimen, respectively. *V*_2_, *m*_2_, and *ρ*_2_ represent the volume, mass, and density of the rock specimen after high-temperature natural cooling.

**Figure 5:**
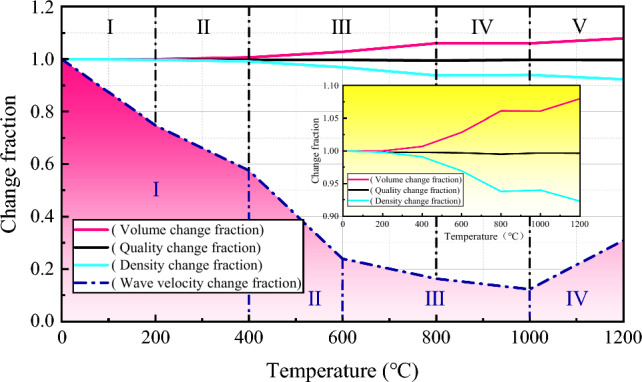
Physical change of granite specimen after heat treatment.

The relationships between various basic physical indicators of granite specimens and temperature changes are shown in Fig. 5. From the overall physical change trend chart, it can be observed that as the temperature increases, the volume change fraction of the granite specimens gradually increases, the density change fraction gradually decreases, and the mass change fraction remains nearly constant. Between 25 and 400 °C, the curves for the volume, mass, and density change fractions are almost coincident, indicating that the physical changes in the granite specimens are not significant within this temperature range. However, from the enlarged local view, it can be seen that at 200 °C, the curves for the volume, mass, and density change fractions begin to show a trend of separation.

Based on the different rates of change in various basic physical indicators, the corresponding change fraction curves can be divided into five stages for detailed comparative analysis, with each analysis using 200 °C as a gradient. In the first stage (25–200 °C), the changes in the volume, mass, and density of the specimens are not significant, with the volume growth rate being only 0.005%, the mass loss rate being 0.198%, and the density loss rate being 0.204%. In the second stage (200–400 °C), the enlarged local view clearly shows that the three curves have transitioned from coinciding to gradually separating, but the change trends are still not obvious. During this stage, the volume growth rate of the specimens has increased from 0.005 to 0.681%, the mass loss rate has only increased from 0.198 to 0.219%, and the density loss rate has increased from 0.204 to 0.894%. The volume expansion detected in this temperature range is mainly due to the expansion of minerals within the rock caused by heating.

In the third stage (400–800 °C), the mass change fraction curve of the specimen is still flat, with the mass loss rate increasing from 0.219% at 400 °C to 0.299% at 600 °C and then to 0.499% at 800 °C. The main reason for the mass loss at 800 °C and before is the escape and evaporation of attached and bound water in the specimens, as well as the pyrolysis of some cementing materials in the specimens^43^. In contrast to the change curve of mass, the change curve of volume has shown a significant upward trend. The volume growth rate increased from 0.681% at 400 °C to 2.85% at 600 °C and then to 6.10% at 800 °C. Similarly, the change curve of density also showed a significant downward trend, with the density loss rate increasing from 0.894% at 400 °C to 3.06% at 600 °C and then to 6.22% at 800 °C. When the heating temperature exceeds 400 °C, both the volume and density of the granite specimens begin to significantly change. This is because the minerals in the granite continue to expand when heated, and the quartz also changes from *α* phase to *β* phase near 573 °C^44^. The volume of quartz rapidly increases after phase transformation, and the process of increasing mineral volume often leads to the generation of numerous microcracks due to extrusion of surrounding mineral crystals. Therefore, starting from 400 °C, the volume growth rate of granite specimens increases significantly with the increase in temperature, and the corresponding density loss rate also increases significantly, resulting in a decrease in overall compactness of the granite specimens.

During the fourth stage (800–1000 °C), the mass loss rate of the specimens decreased modestly from 0.499 to 0.318%. When compared to the mass change fraction curve, both the volume and density change fraction curves, following significant increases, gradually leveled off. Correspondingly, in this stage, the volume growth rate of the specimens slightly decreased from 6.1 to 6.06%, and the density loss rate marginally decreased from 6.22 to 6.01%. In the fifth stage (1000–1200 °C), the mass change fraction curve continued to level off, with the mass loss rate increasing slightly from 0.318 to 0.357%. During this stage, surface spalling became increasingly severe with rising temperatures, manifested by small fragments, particles, and powder detaching from the surface, leading to mass loss. Overall, heat treatment exerted minimal impact on the mass change of the specimens across all stages. From the enlarged local view, the volume and density change fraction curves once again exhibited significant upward and downward trends. The volume growth rate increased from 6.06 to 7.96%, and the density loss rate increased from 6.01 to 7.71%. This phenomenon is attributed to the expansion of minerals within the granite, which macroscopically manifests as local swelling and bulging on the specimen surface, thereby further increasing the volume of the rock specimens and correspondingly decreasing their density.

### Analysis of longitudinal wave velocity variation of granite specimen

The longitudinal wave velocity is an important indicator for assessing rock damage^45^. It is sensitive to the microstructural discontinuities present in rock specimens and can be used to detect the propagation of microcracks. The principle behind this sensitivity is that the wave velocity of ultrasound changes as it passes through different media. If there are structural defects such as cracks or cavities within the rock, the wave velocity will decrease. This study also tested the longitudinal wave velocity to analyze the thermal damage caused by different heating temperatures treatments. The results showed that thermal damage has a significant impact on the longitudinal wave velocity of rocks. The change fraction of longitudinal wave velocity *η*_*Vp*_ is also defined, and the loss rate of longitudinal wave velocity *K*_*Vp*_ is used to characterize the change of longitudinal wave velocity of granite with temperature after high-temperature natural cooling. The loss rate of longitudinal wave velocity is the percentage between the decrease of longitudinal wave velocity of rock specimen after high-temperature natural cooling and the longitudinal wave velocity of original rock specimen. The relationship and calculation formula between the longitudinal wave velocity change fraction and loss rate are shown in Eqs. (7) and (8):

7 $$\eta_{{V_{P} }} = \frac{{V_{P2} }}{{V_{P1} }} \times 100\%$$

8 $$K_{{V_{P} }} = \frac{{V_{P1} - V_{P2} }}{{V_{P1} }} \times 100\% = \left( {1 - \eta_{{V_{P} }} } \right) \times 100\%$$

In the formula above, *V*_*P*1_ is the longitudinal wave velocity of the original rock specimen. *V*_*P*2_ is the longitudinal wave velocity of the cooled rock specimen after high-temperature natural cooling.

According to the different rates of change in the longitudinal wave velocity, the corresponding fractional change curves can be divided into four stages for detailed comparative analysis, as shown in Fig. 5. The analysis is conducted in increments of 200 °C. In the first stage, the fractional change curve of the longitudinal wave velocity shows a steady decline, with a loss of 25.24% from 25 to 200 °C and 17.24% from 200 to 400 °C. In the second stage, the fractional change curve of the longitudinal wave velocity shows a sharp decline, with the wave velocity loss as high as 33.62% from 400 to 600 °C. In the third stage, although the fractional change curve of the longitudinal wave velocity continues to decline, the trend gradually becomes more moderate, with the wave velocity loss of only 7.63% from 600 to 800 °C and 4.05% from 800 to 1000 °C. Notably, among the first three stages, the wave velocity loss of 33.62% from 400 to 600 °C is the highest. The larger the numerical value of the longitudinal wave velocity loss, the more it reflects the sharpness of the decline in the fractional change curve of the wave velocity, and it also indicates the severe deterioration of the physical and mechanical properties of the rock specimens at the macroscopic level. Additionally, the slope of the fractional change curve of the longitudinal wave velocity starts to decrease at 600 °C, indicating that the extent of the wave velocity loss begins to slow down from 600 °C. Therefore, 600 °C can be considered the threshold temperature for the longitudinal wave velocity loss in the granite used. The sharp reduction in the wave velocity of granite specimens at this temperature gradient is related to the development, propagation, and interconnection of numerous microcracks within the rock specimens. The development of a large number of microcracks signifies an increase in internal discontinuities, thereby significantly reducing the wave velocity^12–14^.

**Figure 6:**
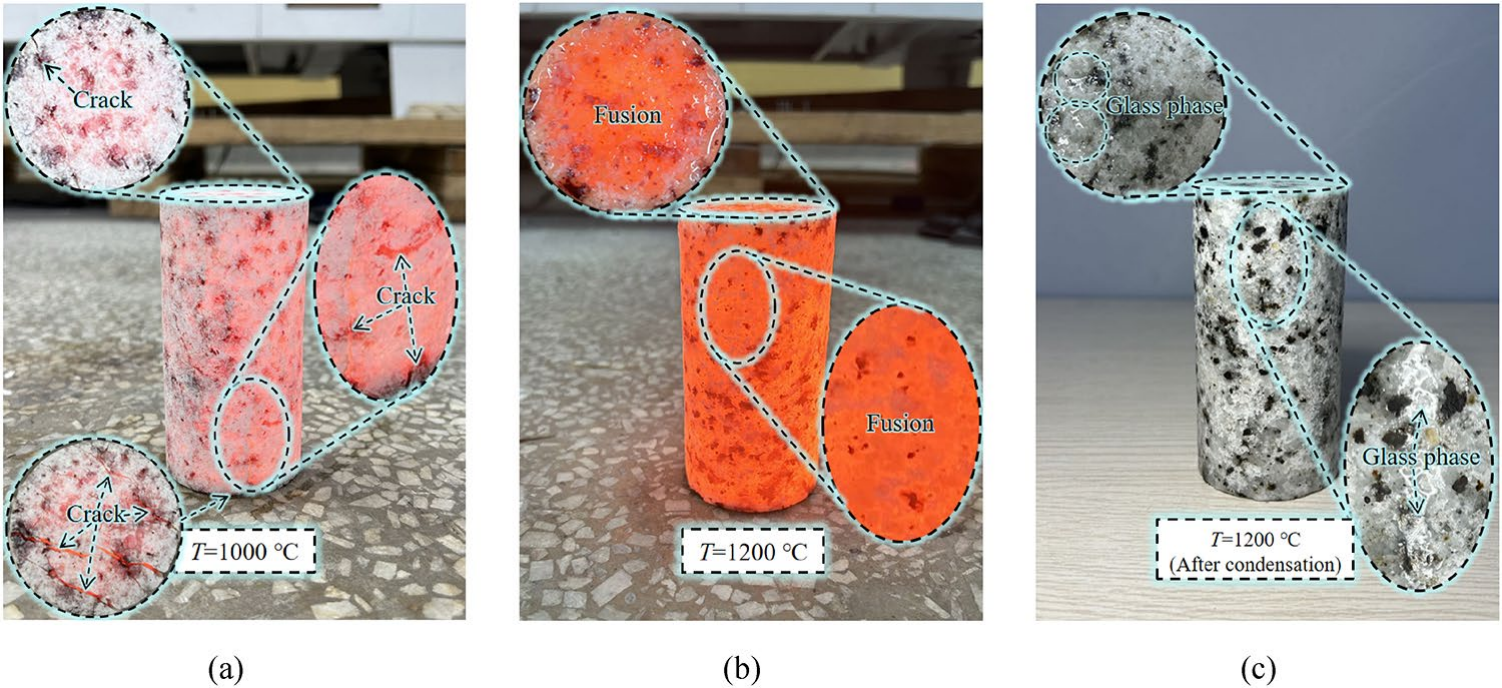
Granite specimens at 1000 °C and 1200 °C. (**a**) T = 1000 ℃ (**b**) T = 1200 ℃ (**c**) T = 1200 ℃.

During the fourth stage, when the temperature ranged from 1000 to 1200 °C, there was a certain degree of rebounding trend in the change fraction curve of longitudinal wave velocity. Correspondingly, the wave velocity increased by 18.72% during this temperature range. This is because at temperatures between 1000 and 1200 °C, various minerals within the granite gradually reached their melting critical values, and after cooling and solidifying into a glassy phase, some internal structural defects disappeared. The reduction in the number of microcracks is manifested in the rebounding of longitudinal wave velocity. Specifically, the velocity increased from 398 m/s at 1000 °C to 955 m/s at 1200 °C. This is because the longitudinal wave velocity is highly sensitive to the discontinuity of rock specimens. The decrease in crack numbers implies an enhancement in continuity, hence the rebounding of longitudinal wave velocity. Granite specimens heated to 1000 °C and 1200 °C are shown in Fig. 6, where obvious cracks are observed on the surface of the specimen at 1000 °C, but no cracks are observed on the surface of the specimen after cooling and solidifying into a glassy phase at 1200 °C. The above content reflects the degradation pattern of rock matrix integrity with the increase in heating level.

### Analysis of internal microcrack propagation and evolution laws in granite

**Figure 7:**
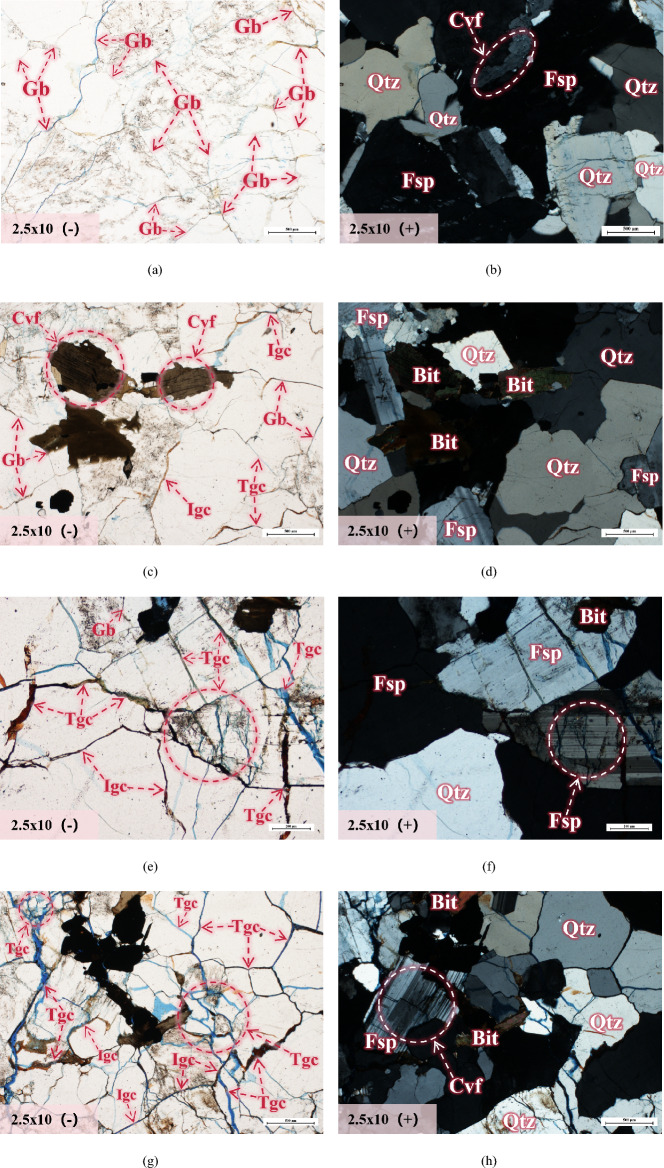

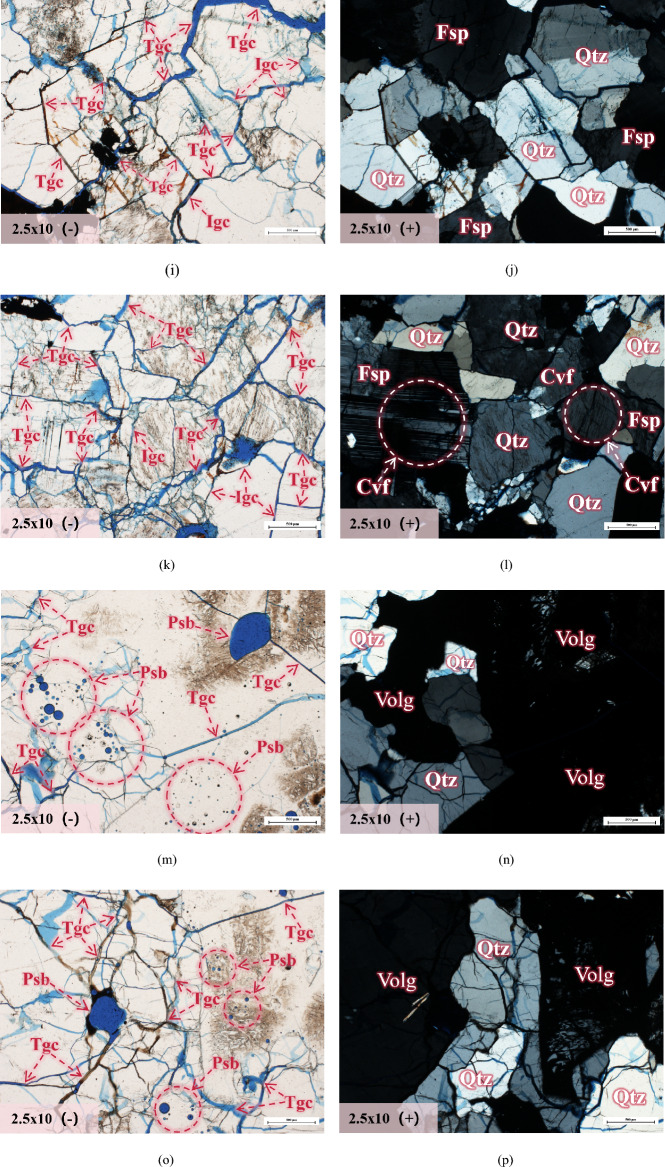
Microstructure and mineral characteristics of granite after different temperature treatments (part). All microstructures are magnified by 2.5 × 10 times, and the scale length in the lower right corner of the photo is 200 μm. The single polarized light photos are indicated with (−), mainly used for the analysis of microstructural features of granite (Gb-grain boundary, Igc-intergranular crack, Tgc-transgranular crack, Cvf-cleavage fracture, Psb-Pearl-shaped bubbles). The orthogonal polarized light photos are indicated with (+), mainly used for the analysis of mineral characteristics of granite (Qtz-quartz, Fsp-feldspar, Bit-biotite, Volg-volcanic glass). It should be noted that the single polarized light and orthogonal polarized light photos under different temperature gradients should be used in conjunction for comparative observation analysis, as they observe the same position. (**a**) T = 25 ℃ (**b**) T = 25 ℃ (**c**) T = 200 ℃ (**d**) T = 200 ℃ (**e**) T = 400 ℃ (**f**) T = 400 ℃ (**g**) T = 600 ℃ (**h**) T = 600 ℃ (**i**) T = 800 ℃ (**j**) T = 800 ℃ (**k**) T = 1000 ℃ (**l**) T = 1000 ℃ (**m**) T = 1200 ℃ (**n**) T = 1200 ℃ (**o**) T = 1200 ℃ (**p**) T = 1200 ℃.

In order to further investigate the evolution law of internal microcracks in granite specimens with temperature, we also used a research-grade polarizing microscope to perform single-polarized and orthogonal-polarized observation and analysis on the casting thin sections of the specimens. The microstructures inside the specimens were magnified by 2.5 × 10 times, and the results are shown in Fig. 7.

According to Fig. 7a and b, it can be seen that the minerals within the granite are tightly interconnected at room temperature, and there is no separation between grain boundaries. Cleavages can be observed within the feldspar. At 200 °C, the mineral grain boundaries start to separate, and both intergranular and intragranular cracks appear. The presence of cleavage can also be observed in feldspar and biotite, as shown in Fig. 7c and d. At 400 °C, the grain boundaries almost disappear and evolve into intergranular cracks. Transgranular cracks begin to develop and penetrate, resulting in transgranular cracks between the grains as shown in Fig. 7e and f. At 600 °C, the grain boundaries completely disappear, and a large number of transgranular cracks emerge, intertwining with intergranular cracks to form a crack network, as shown in Fig. 7g and h. From Fig. 7i and j, it can be observed that at 800 °C, the internal damage of the rock becomes severe, with more significant development of intergranular cracks and transgranular cracks, and a more complex structural morphology. Figure 7k and l demonstrates that at 1000 °C, the internal damage of the rock further intensifies, and various types of cracks continue to develop, forming a denser network of through-cracks. At 1200 °C, all major minerals within the granite have reached their melting point. Most of the black opaque areas can be observed through Fig. 7m and p, which is volcanic glass formed by the melting and solidification of minerals within granite^46,47^, It is worth noting that the number of cracks significantly decreases within the volcanic glass region, and numerous pearl-like bubbles of varying sizes can be clearly observed.

### Analysis of mineral changes of granite specimens

#### Analysis of XRF and microscopic mineral characteristics

The Axios-Max (PW4400/40) wavelength dispersive X-ray fluorescence spectrometer was used to analyze the X-ray fluorescence spectroscopy (XRF) of granite treated after different temperatures. The content of major elements is shown in Table 2. It can be seen that the influence of temperature on the change in the content of each element in the rock is not obvious, but it also confirms from the side that the granite used in the experiment has strong homogeneity.

**Table 2:** Contents of chemical elements in granite treated at different temperatures.

Temperature/ (℃)	25	200	400	600	800	1000	1200
O (%)	39.49	37.78	32.73	39.73	30.38	36.65	35.05
Na (%)	2.64	2.72	2.82	2.68	2.53	2.62	2.54
Mg (%)	0.29	0.19	0.14	0.16	0.19	0.11	0.13
Al (%)	6.73	6.70	7.13	6.60	6.60	6.90	6.34
Si (%)	30.30	31.66	31.02	31.88	31.19	32.46	30.88
P (%)	0.02	0.01	0.02	0.02	0.02	0.01	0.01
K (%)	3.80	3.76	3.99	3.93	4.10	4.22	4.09
Ca (%)	2.61	1.50	1.13	1.06	1.39	1.03	1.53
Fe (%)	0.50	0.41	0.52	0.48	0.46	0.50	0.51
Total content (%)	86.38	84.73	79.5	86.54	76.86	84.5	81.08

Percentage by mass is used here to represent the element content.

The main minerals in the granite used in the experiment were determined by XRD to be quartz (SiO_2_), potash feldspar (KAlSi_3_O_8_), plagioclase (Na [AlSi_3_O_8_]–Ca[Al_2_Si_2_O_8_]), and a small amount of biotite (K (Mg, Fe^+2^)_3_ (AlSi_3_O_10_) (OH)_2_). The relative content of the main mineral components in the rock is calculated using the K-value method based on Eq. (9)^48^. Table 3 summarizes the changes in the internal mineral content of the granite after treatment at different temperatures. In the undamaged granite, plagioclase accounts for 47.1%, potash feldspar accounts for 18.7%, quartz accounts for only 25.2%, and biotite accounts for only 7.4%.

9 $$W_{X} = \frac{{I_{X} }}{{K_{A}^{X} \sum_{i = A}^{N} \frac{{I_{i} }}{{K_{A}^{i} }}}}$$

**Table 3:** Characteristic parameters of granite mineral composition after different temperature treatment.

Temperature/ (℃)	25	200	400	600	800	1000	1200
Plagioclase feldspar (%)	47.1%	45.3%	43.3%	42.9%	36.6%	31.2%	0%
Potash feldspar (%)	18.7%	23.1%	19.7%	23.4%	28.6%	36.3%	0%
Quartz (%)	25.2%	24.3%	26.5%	26.3%	27.4%	29.1%	21.2%
Biotite (%)	7.4%	6.4%	7.3%	5.2%	4.1%	2.8%	0%
Other minerals (%)	1.6%	0.9%	3.2%	2.2%	3.3%	0.6%	78.8%

The other minerals at 1200 °C are volcanic glass.

In the formula: *W*_*X*_ is the relative content of phase X of something, *I*_*X*_ is the diffraction intensity of something phase X, A is the name of the phase selected as the internal standard phase in N phases, $K_{A}^{X}$ is the ratio of RIR value between phase X and internal standard phase A.

Based on Table 3, due to the presence of (OH)^−^ and natural cleavage in biotite minerals, the thermal decomposition of biotite begins at 200 °C,From Fig. 7c and d, the cracks derived from cleavage within biotite can be clearly observed. At 600 °C, the thermal decomposition of biotite is enhanced, and it undergoes a rapid expansion perpendicular to the cleavage surface^11^. At 1000 °C, the biotite content decreases by 3.6%, decomposing into magnetite (Fe_3_O_4_) and garnet, and undergoes melting and solidification to form a glass phase at 1200 °C. Compared to biotite, the content of plagioclase remains relatively stable from 25 to 600 °C and shows a slight downward trend. However, at 800 °C, the content of plagioclase decreases significantly, from 42.9% at 600 °C to 36.6% at 800 °C. At 800 °C to 1000 °C, potash feldspar begins to grow significantly, from 28.6% at 800 °C to 36.3% at 1000 °C. This is closely related to the thermal decomposition of plagioclase (Na [AlSi_3_O_8_]). Na and K ions are replaced with each other in plagioclase and potash feldspar decomposed under high temperature, which is the potassium feldsparization in the pyrolysis process^24^. However, the potash feldspar that showed a significant growth trend from 800 °C has already transformed into its homomorphic polymorph: anorthoclase ([Na, K][Si_3_Al]O_8_). Correspondingly, its structure has also changed, the specific manifestation is from the monoclinic crystal system with strong symmetry to the triclinic crystal system with poor symmetry, which leads to the decrease of structural stability.

The evolution of the microscopic molecular structure of various minerals with temperature plays a crucial role in explaining the thermal damage mechanism of granite. The fundamental reason for the thermal decomposition of feldspar minerals lies in the fracture of their microscopic molecular chemical bonds. The chemical bonds of feldspar minerals are mainly composed of ionic bonds and covalent bonds^49^. Here, ionic bonds refer to the chemical bonds between sodium ions and oxygen ions, and potassium ions and oxygen ions, respectively, while covalent bonds refer to the chemical bonds between aluminum ions and silicon ions with oxygen ions. The fracture temperature of these chemical bonds is an important physical property of feldspar minerals, which determines the stability of feldspar minerals under high temperatures. As feldspar is the main mineral in the granite studied this time, its stability also determines the stability of thermally damaged granite. The fracture temperature of chemical bonds refers to the energy absorbed by the fracture of chemical bonds under certain conditions. Chemical bonds are essentially a type of force, and high temperatures provide energy to them. Therefore, after absorbing energy, the chemical bonds in feldspar molecules fracture, leading to the thermal decomposition of feldspar minerals and promoting the growth of unstable mineral anorthoclase.

**Figure 8:**
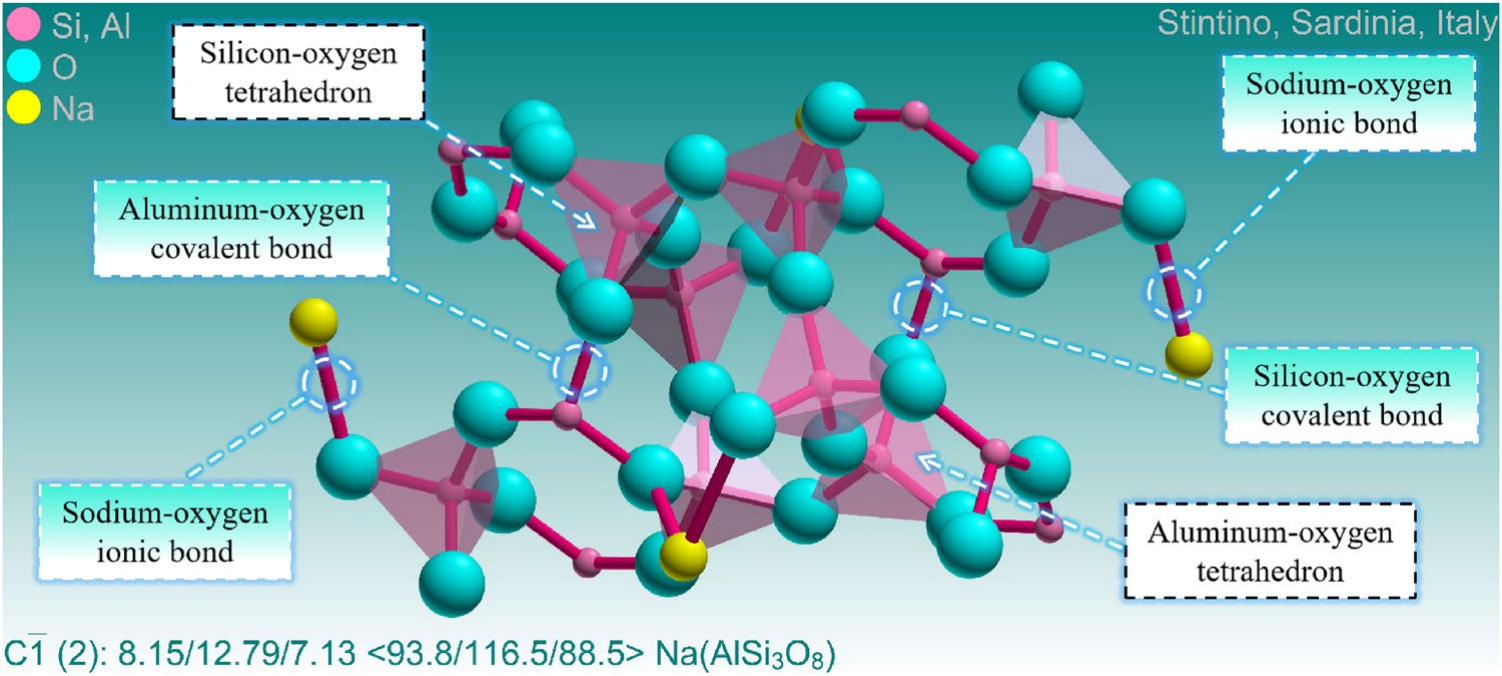
Microscopic molecular structure of albite.

**Figure 9:**
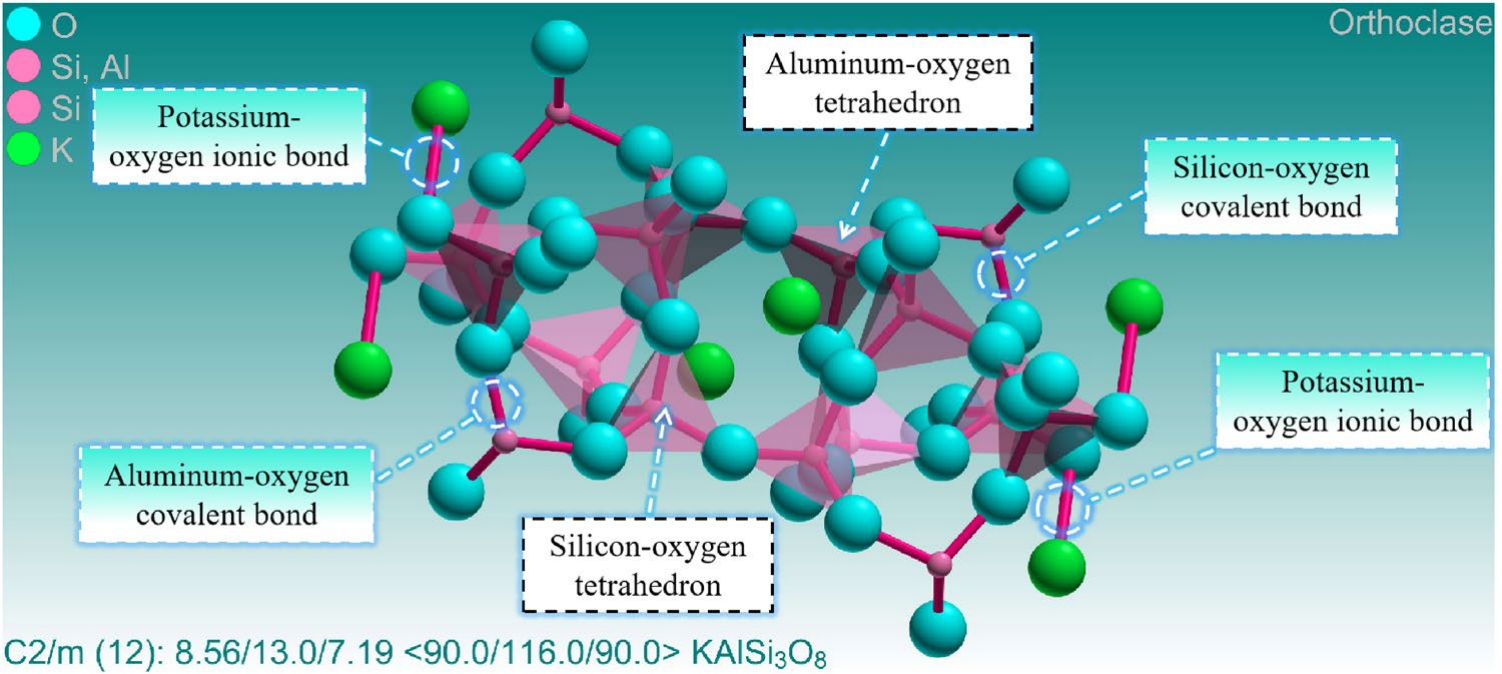
Microscopic molecular structure of potash feldspar.

The generation of new substances is an important basis for determining the formation and breakage of chemical bonds, which coincides with the significant decrease in albite content detected at 800 °C and the rapid growth of anorthoclase. However, the temperatures at which different chemical bonds break are quite different. Generally speaking, the breaking temperature of ion bonds in feldspar minerals is about 800 °C, while the breaking temperature of covalent bonds is about 1200 °C. This is because the breaking temperature of chemical bonds in feldspar minerals is closely related to their crystal structure. The basic structure of feldspar is a stable tetrahedral structure, mainly composed of one silicon or aluminum atom carrying four oxygen atoms. The tetrahedra can also share oxygen atoms with each other and then achieve a connection through silicon-oxygen covalent bonds and aluminum-oxygen covalent bonds, forming the framework structure of feldspar minerals^50–53^. Although both are covalent bonds, the breaking temperature of silicon-oxygen covalent bonds is usually higher than that of aluminum-oxygen covalent bonds because silicon-oxygen covalent bonds are stronger than aluminum-oxygen covalent bonds. The microstructure of albite and potash feldspar are shown in Figs. 8 and 9 respectively. In addition, the breaking temperature of chemical bonds in feldspar minerals is also influenced by their chemical composition. For example, when the sodium content in feldspar minerals increases, the breaking temperature of chemical bonds decreases. This is because sodium ions are larger than potassium ions, resulting in an increase in the looseness of the crystal structure and making the chemical bonds easier to break. Studies have found that the crystallization and thermal expansion of plagioclase have led to changes in the internal structure of granite, which is also an important factor that affects the mechanical properties of granite from a microscopic perspective^54,55^. As shown in Fig. 7f, h, and l, it can be observed that natural cleavage exists within the plagioclase. Under the influence of temperature, chemical bonds in the plagioclase molecules are broken, leading to the formation of numerous transgranular cracks. In general, the temperature at which the chemical bonds in the plagioclase fracture is a relatively complex issue that requires considering multiple factors. Further research in the future will continue to explore this topic in depth.

**Figure 10:**
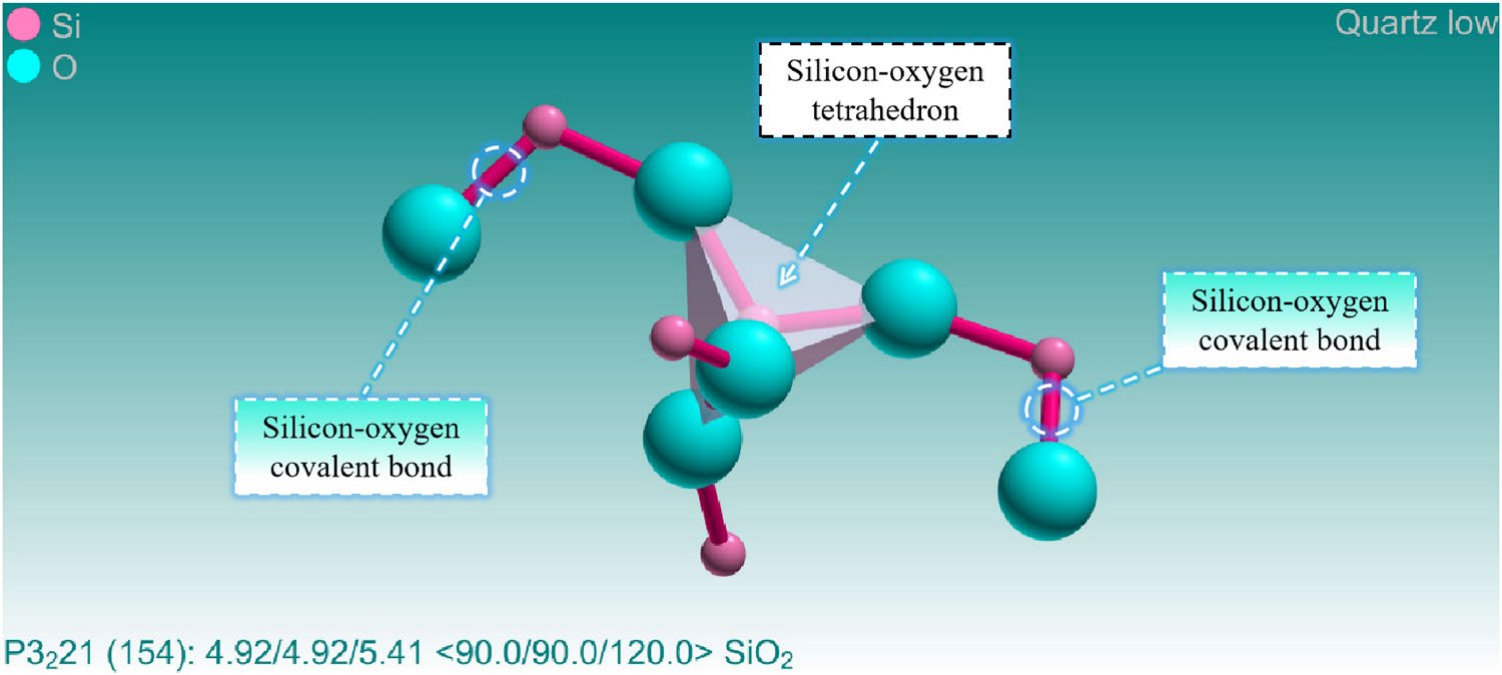
Microscopic molecular structure of quartz.

According to Table 3, it can be seen that the quartz content varies within a range of 4.8% from 25 to 1000 °C, indicating that high temperature has little effect on the quartz content. From 1000 to 1200 °C, the quartz content decreases by 7.9%, which is related to the formation of glassy phase after the melting and solidification of various minerals (including some quartz) in the granite at 1200 °C. Although high temperature has little impact on the quartz content, quartz undergoes a polymorphic transformation at high temperatures, leading to a significant increase in volume, as shown in Table 4^25^. According to the reactions in the table, it can be concluded that the original volume of quartz has increased by 16% at 870 °C. From Fig. 7, it can be observed that compared to biotite and feldspar minerals, quartz does not have natural cleavage and exhibits higher stability. This is because the silicon-oxygen tetrahedra in quartz are connected in six-membered ring structures, and the chemical bonds between them are silicon-oxygen covalent bonds^56^, thereby providing high stability to the quartz crystal structure. On the other hand, feldspar is composed of tetrahedral chains, which are then interconnected to form a framework structure. Generally, the interchain connections in feldspar are relatively weak, resulting in the occurrence of structural defects in crystallographic arrangements. In addition, there are important differences between the silicon-oxygen tetrahedra formed within quartz molecules and the silico-aluminum tetrahedra within feldspar molecules. Furthermore, feldspar molecules contain metal cations such as potassium, calcium, and sodium, which weaken the surrounding silicon-oxygen bonds due to the ionic bonds formed between these cations and oxygen ions. Therefore, compared to feldspar minerals, quartz possesses better thermal stability. The microstructure of quartz is shown in Fig. 10. From Fig. 7c–f, it can be observed that only a small number of transgranular cracks exist within quartz at temperatures ranging from 200 to 400 °C. At temperatures from 600 to 1200 °C, the number of transgranular cracks in quartz significantly increases, and their morphological structures become more complex, as shown in Fig. 7g–p. It is worth noting that the number of cracks inside the grains of feldspar and biotite is significantly higher than that within quartz under the same temperature gradient. Additionally, while all minerals reach their melting points and condense into volcanic glass at 1200 °C, quartz still exists due to its higher melting point.

**Table 4:** Quartz phase transition and percentage volume increase at different temperatures.

Temperature/ (℃)	Reaction
117	*α*—tridymite ⇋ *β*_*1*_—tridymite + 0.2%
163	*β*_*1*_—tridymite ⇋ *α*—tridymite + 0.2%
180–270	*β*—cristobalite ⇋ *α*—cristobalite + 2.0%
573	*β*—quartz ⇋ *α*—cristobalite + 0.82%
870	*α*—quartz ⇋ *α*—tridymite + 16%
1000	*α*—quartz ⇋ *α*—cristobalite + 15%

**Figure 11:**
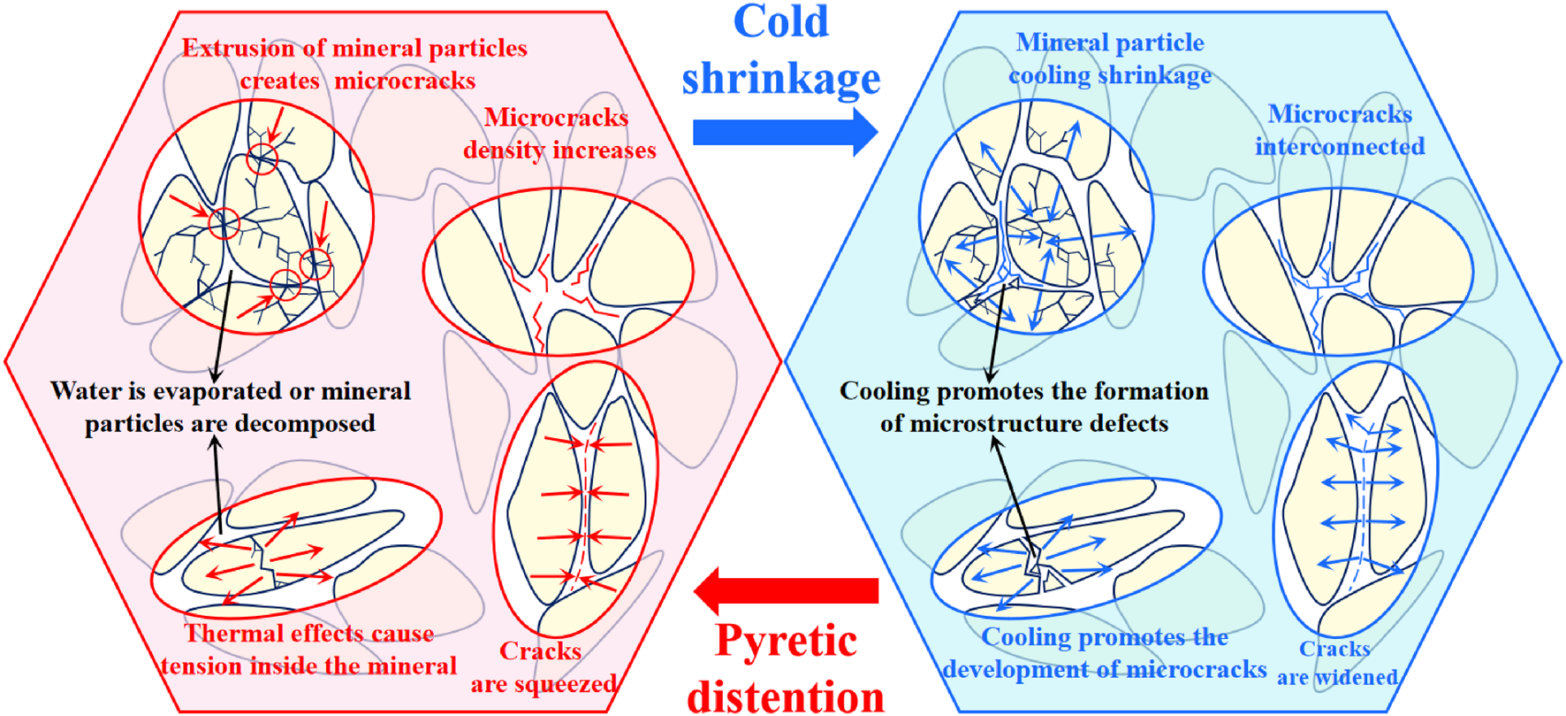
Microscopic thermal damage mechanism of rock in the process of heating and cooling.

Thermal fracturing is one of the significant causes of thermal damage in granite. The temperature stress generated at high temperatures disrupts the original cementation structure between mineral particles, leading to the generation of numerous microcracks within the rock specimen. As a result, overall density decreases and internal damage intensifies. Additionally, granite is composed of various mineral particles, differing in size, shape, degree of interparticle bonding, porosity, pore structure, expansion coefficient, thermal elastic properties, and condensation rate. Therefore, the combined structural thermal stress arising from the thermal expansion and cooling contraction processes of different mineral particles promotes the development of microcracks and further exacerbates internal rock damage, The microscopic thermal damage mechanism of rock is shown in Fig. 11. Thermal activation is another important factor contributing to thermal damage in granite. The thermal motion or stress action of rock crystal particles can cause dislocations in rock crystals, making them prone to fracture. Research on quartz crystals has revealed that after experiencing high temperatures of 400 °C, –OH radicals within the crystals are thermally activated, causing the substitution of some silicon-oxygen bonds with hydroxyl groups. This facilitates the formation of internal microflaws such as dislocations within mineral crystals in granite, consequently weakening the strength of specimens ^57^.

In summary, the mineral composition, intergranular cementation structure, microstructure, and even chemical composition within granite will undergo varying degrees of changes with temperature in a high-temperature environment. The mineral composition, development of microstructure defects, and the degree of interconnection between mineral particles determine the mechanical properties of granite from a microscopic perspective.

#### XRD phase characteristic analysis

**Figure 12:**
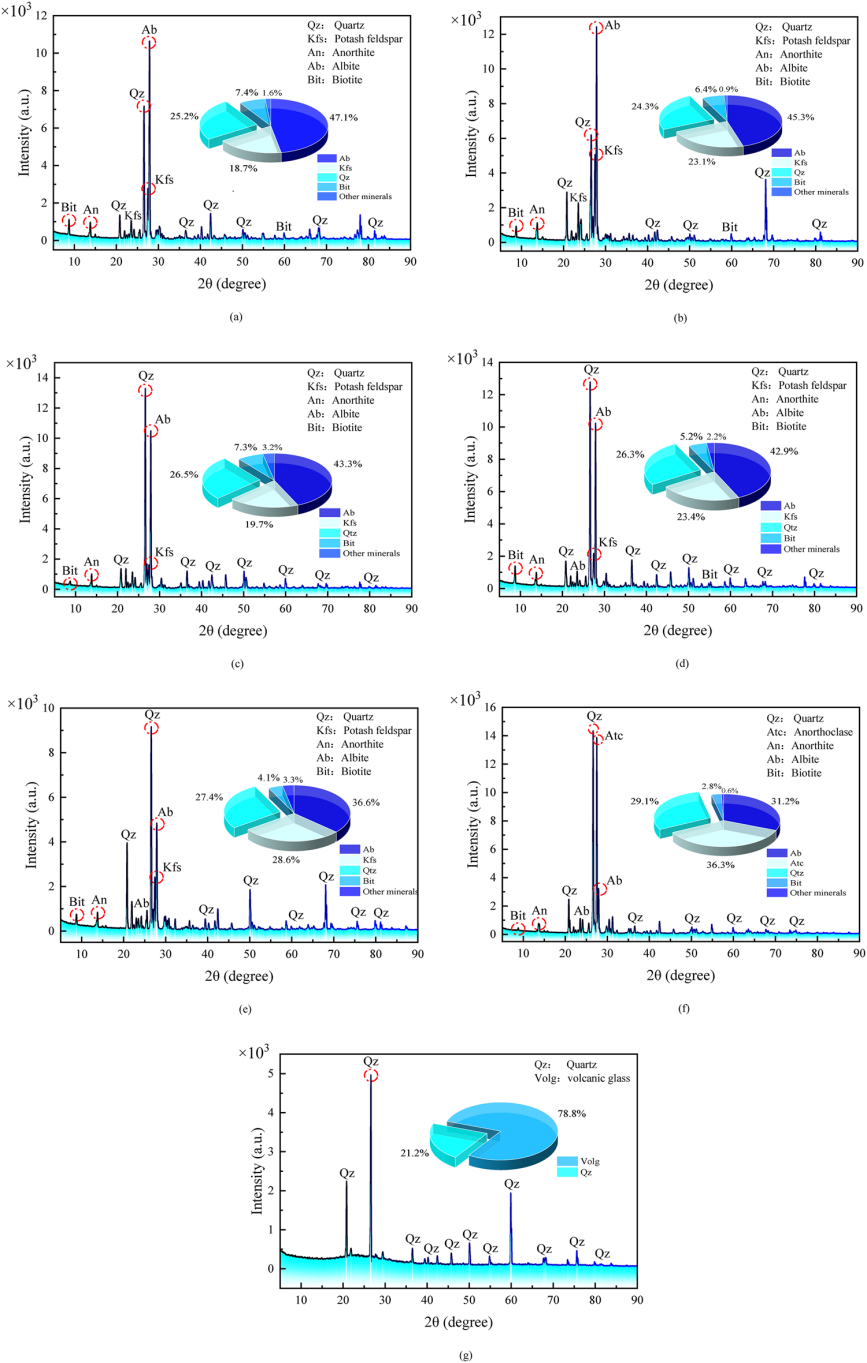
Mineralogical characteristics of granite after different temperatures treatment (XRD diffraction pattern and mineral proportion). (**a**) T = 25 ℃ (**b**) T = 200 ℃ (**c**) T = 400 ℃ (**d**) T = 600 ℃ (**e**) T = 800 ℃ (**f**) T = 1000 ℃ (**g**) T = 1200 ℃.

The XRD phase characteristics of granite treated at different temperatures were analyzed using the X’Pert3 Powder multi-purpose powder X-ray diffraction instrument, which helps further analyze the relationship between different mineral components and their basic properties in the granite specimens with temperature variations. This will reveal the thermal damage mechanism of high-temperature granite from a microscopic perspective. The XRD diffraction patterns of granite powder samples after treatment at different temperatures are shown in Fig. 12a–g.

By comparing and analyzing the mineral diffraction peaks of granite treated at different temperatures, it can be concluded that there are no significant changes in the main mineral components of granite between 25 and 800 °C. The specific diffraction information of various minerals also did not show obvious changes, which include biotite, anorthite, potash feldspar, albite, and quartz. This indicates that the main mineral components in the granite are relatively stable within this temperature range. In contrast, it can be observed from Fig. 12e that the diffraction peak of albite significantly decreases at 800 °C. Similarly, at 1000 °C, the diffraction peak of potash feldspar disappears, and it is replaced by the diffraction peak of anorthoclase, as shown in Fig. 12f. Finally, according to Fig. 12g, only the diffraction peak of quartz can be detected at 1200 °C. It should be noted that the diffraction information of weak peaks cannot accurately reflect the variation of basic properties of various minerals in granite with temperature. Therefore, characteristic peaks of each type of mineral circled in red in Fig. 12a–g are selected for analysis to ensure the representativeness, comparability, and accuracy of the data. Refer to Table 5 for detailed parameters of characteristic diffraction peaks.

**Table 5:** Characteristic diffraction peak parameters of main minerals in granite samples treated at different temperatures.

*T* (℃)	Quartz		Albite		Anorthite		Potash feldspar		Biotite	
	Intensity (CPS)	FWHM (2*θ*)	Intensity (CPS)	FWHM (2*θ*)	Intensity (CPS)	FWHM (2*θ*)	Intensity (CPS)	FWHM (2*θ*)	Intensity (CPS)	FWHM (2*θ*)
25	7171	0.161	10,643	0.152	971	0.185	2773	0.126	1109	0.156
200	6193	0.172	12,421	0.141	1128	0.223	5058	0.129	923	0.160
400	13,287	0.141	10,484	0.130	1013	0.162	1620	0.132	535	0.189
600	12,781	0.148	10,231	0.123	988	0.184	2162	0.137	1393	0.163
800	9167	0.136	4844	0.133	842	0.167	2423	0.130	759	0.157
1000	14,336	0.126	3235	0.112	729	0.169	13,880	0.121	456	0.125
1200	4964	0.129	None	None	None	None	None	None	None	None

The changes in mineral composition and basic properties of granite with temperature are characterized using diffraction peak intensity and full width at half maximum (FWHM). The diffraction peak intensity reflects not only the content of mineral components but also the crystallinity of various mineral crystals. Larger peak intensity indicates better crystal development and higher crystallinity. On the other hand, FWHM represents the grain volume of different mineral crystals. A larger FWHM value indicates a wider diffraction peak and a relatively smaller grain volume, while a smaller FWHM value indicates a sharper diffraction peak and a relatively larger grain volume. It should be noted that the mineral powder particles used for diffraction analysis are extracted from naturally cooled granite specimens. The grain volume of each mineral in the naturally cooled granite specimens is somewhat contracted compared to the high-temperature state but cannot fully recover to the original size. This is because the mineral grains undergo deformation during the thermal expansion and contraction process, and some of the deformation is irreversible plastic deformation. However, the volume of mineral grains is larger in actual high-temperature environments.

The results show that there are two characteristic temperature points of 400 °C and 1200 °C for the variation of quartz diffraction intensity. At 400 °C, the diffraction intensity of quartz began to increase significantly, and the increase was nearly twice that at 200 °C, and then the diffraction intensity of quartz remained in a very high range at 400–1000 °C. This is related to the rapid increase of quartz grain volume after the transformation, and the original volume of quartz has increased by 16% at 870 °C. At 1200 °C, the diffraction intensity of quartz began to decrease significantly, and the quartz began to decompose. From 0.161 at 25 °C to 0.129 at 1200 °C, the FWHM parameter value of quartz decreases continuously, which also indicates that the quartz grain volume increases continuously. In general, the better the crystalline state of a crystal, the larger the grain size, and the higher the intensity of diffraction peaks. The increase in quartz grain size is an important factor contributing to the significant enhancement of its diffraction intensity. In contrast to quartz, the diffraction intensity of feldspar minerals generally shows a decreasing trend. In particular, the diffraction intensity of albite decreases from 10,231 at 600 °C to 4844 at 800 °C, which is associated with the rapid decomposition of albite upon reaching its threshold temperature for thermal decomposition. Decomposition of materials often accompanies the generation of new substances. Therefore, a significant increase in diffraction intensity of potash feldspar can also be detected at 1000 °C. However, by this time, the potash feldspar that started to significantly increase its diffraction intensity has already transformed into its poorly-property homomorphic polymorph anorthoclase. On the other hand, the FWHM parameter values of albite show a continuous decreasing trend, while the FWHM parameter values of anorthite and potash feldspar have unstable changing trends. Nevertheless, comparing the parameter values at 25 °C and 1000 °C, both anorthite and potash feldspar show varying degrees of decrease, indicating that the volume of different types of feldspar minerals has correspondingly increased.

It can be observed that the diffraction intensity of biotite is abnormally high at 600 °C, but overall, the diffraction intensity is still decreasing continuously. This phenomenon is related to the thermal decomposition of biotite. Starting from 400 °C, the FWHM parameter value of biotite continuously decreases, indicating the continuous expansion of mineral particles. In conclusion, the decrease in diffraction peak intensity signifies the decomposition of stable minerals, a decrease in crystallinity, and a loosening of crystal structure. The decrease in the FWHM parameter value indicates an increase in the volume of mineral grains, which not only means the destruction of the original cementation structure between mineral grains but also leads to the compression of surrounding crystals, promoting the development of microcracks. Minerals with natural cleavage structure defects such as feldspar and biotite will rapidly develop internal cleavage as their mineral grain volume increases, playing a crucial role in the propagation of microcracks within rocks.

In summary, the changes in mineral composition and basic properties of rocks caused by high temperatures are the main reasons for the damage to rock materials by high temperatures. As the temperature continues to rise, the feldspar minerals in granite will decompose into clay minerals such as kaolinite, illite, and montmorillonite. At the same time, the decomposition of albite and potassium feldspar promotes the formation of anorthoclase, leading to a decrease in structural stability. Although quartz has relatively stable properties, it also undergoes a phase transition at around 573 °C, resulting in significant changes in its crystal structure. With the increase in the unstable mineral content within the rock and the development of crystal structure defects in various minerals, the mechanical properties of granite specimens deteriorate sharply at the macroscopic level, while a decrease in brittleness and an increase in ductility are observed.

### Analysis of stress–strain behaviors of granite specimens

**Figure 13:**
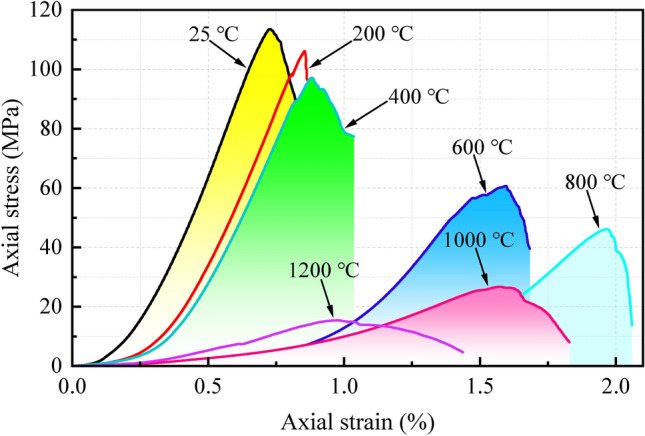
Stress–strain curves of granite specimens treated at different temperatures.

Thermal damage significantly affects the stress–strain relationship prior to peak stress. The stress–strain curves of granite subjected to damage at different temperatures are shown in Fig. 13. Granite is a typical brittle rock, exhibiting brittle failure under uniaxial compression at room temperature. With the increase in heating temperature, the characteristics of its brittle failure gradually weaken.

As the temperature increases, the initial deformation stage of the stress–strain curve gradually becomes gentle, and the crack closure and compaction stage of the rock specimens is prolonged. The temperature of 600 °C is the characteristic point where the stress–strain curve undergoes significant changes. At this point, the initial deformation stage of the stress–strain curve almost tends to be flat, and the crack closure and compaction stage significantly prolongs. Similarly, the axial strain detected during this stage also increases significantly. At 800 °C, the trend of the curve for the crack closure and compaction stage of the granite specimens becomes more pronounced, and correspondingly, the crack closure and compaction stage continues to extend. This is related to the occurrence of numerous micro-cracks and structural defects within the rock specimens at 800 °C. In contrast, the slope of the crack closure and compaction stage curve of the stress–strain curve increases at 1000 °C, and this phenomenon becomes more evident at 1200 °C. This is because various minerals inside the granite specimens gradually reach their melting points between 1000 and 1200 °C. After condensation, the rock specimens not only exhibit glass brittleness but also witness the disappearance of some internal structural defects such as micro-cracks and pores. This corresponds to the phenomenon of rebound in longitudinal wave velocity of the granite specimens at 1000–1200 °C. The post-peak behavior of the granite specimens is also significantly influenced by temperature. When the granite specimens are not subjected to thermal damage, their post-peak stress–strain curve shows brittle behavior. That is, when the peak stress is reached, the strength rapidly decreases to residual. However, as the temperature increases, the stress–strain curve exhibits softening behavior, and the post-peak stage gradually extends and tends to be gentle, enhancing the ductility of the rock material. In summary, thermal damage enhances the ductility of the granite specimens. Detailed results of mechanical tests can be found in Table 6.

**Table 6:** Summary of experimental results of granite specimens after treatment at different temperatures.

*T* (℃)	*V*_*P*_ (m·s^-1^)	*UCS* (MPa)	*E* (GPa)	*ε*_*p*_ (%)	Failure mechanism	Microscopic fracture pattern	Failure mode
25	3079	113.54	20.28	0.73	Sudden instability	Intergranular crack	Splitting
200	2302	106.14	17.66	0.86	Sudden instability	Intergranular and intragranular cracks	Splitting
400	1693	97.12	17.39	0.88	Sudden instability	Transgranular crack	Splitting
600	720	60.64	8.26	1.60	Quasi-sudden instability	Transgranular crack	Splitting
800	493	46.09	4.13	1.96	Progressive instability	Through-crack network	Splitting
1000	398	26.71	2.25	1.58	Progressive instability	Through-crack network	conical
1200	955	15.41	1.88	0.98	Progressive instability	Transgranular crack	Splitting

*V*_*P*_—longitudinal wave velocity, *E*—elastic modulus, *ε*_*p*_—axial strain corresponding to peak strength.

**Figure 14:**
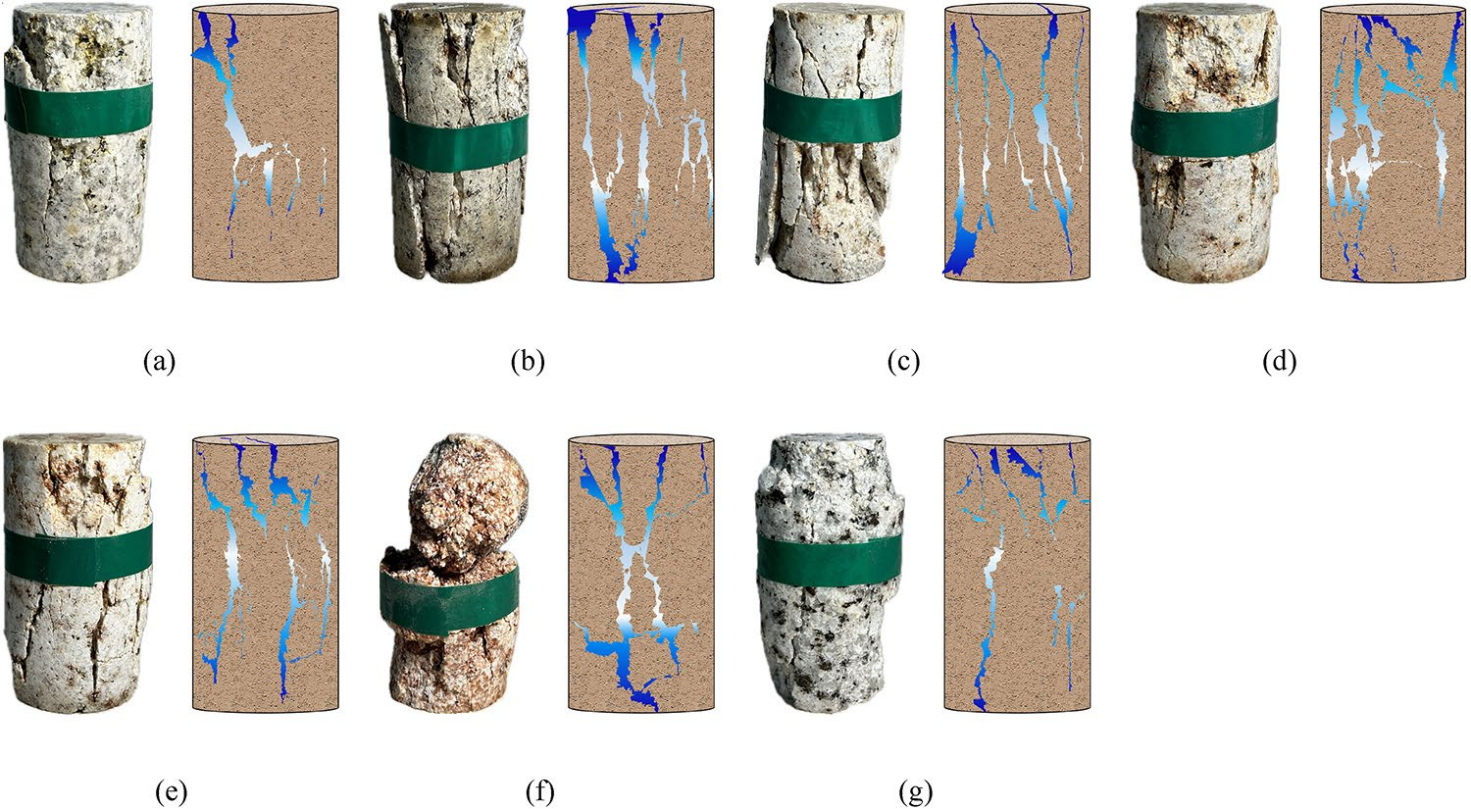
The failure mode of granite specimens after different temperature treatment. (**a**) T = 25 ℃ (**b**) T = 200 ℃ (**c**) T = 400 ℃ (**d**) T = 600 ℃ (**e**) T = 800 ℃ (**f**) T = 1000 ℃ (**g**) T = 1200 ℃.

Figure 14 demonstrates that the failure modes of heat-damaged granite specimens are generally similar, Apart from the conical-shaped failure observed at 1000 °C, the rest exhibit a mode of splitting failure. It is worth noting that with 600 °C as the dividing point, the granite specimens showed sudden instability failure before 600 °C, and the damage sounded loudly, which indicated that a huge elastic potential energy was accumulated in the specimens within this temperature range, making the specimens brittle and hard. After 600 °C, the granite specimen turns into progressive instability failure, and the failure sound is relatively calm, which indicates that the rock specimen accumulates less elastic energy before failure, and the brittleness of the specimen is weakened and the ductility is enhanced.

**Figure 15:**
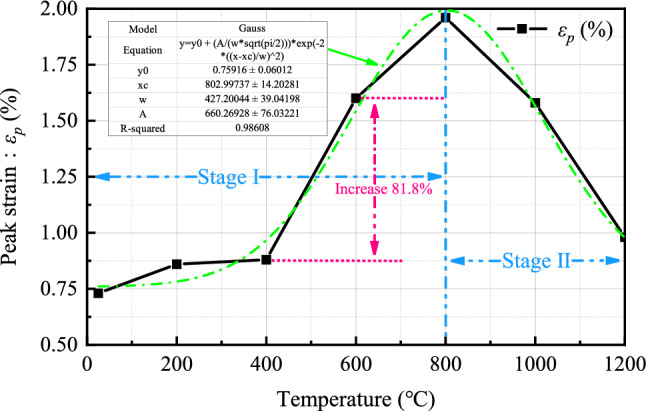
The variation of peak strain (*ε*_*p*_) with temperature.

The axial strain (*ε*_*p*_) corresponding to peak stress increases first and then decreases with the increase in temperature, as shown in Fig. 15. According to the different trends in the curve, it is divided into ascending and descending stages for analysis. The appearance of two distinctly different stages fundamentally reflects the two opposite processes of thermal damage granite specimens experiencing a transition from brittleness to ductility and then back to brittleness. During the ascending stage (25–800 °C), the peak strain of the specimen continuously increases, indicating a transition from brittleness to ductility. This is related to the derivation of numerous microcracks within the specimen, the transformation of mineral properties, and the destruction of the cementing structure. The destruction of the cementing structure implies a significant weakening of the rock’s resistance to deformation, thus enhancing the ductility of the specimen. During the descending stage (800–1200 °C), the peak strain of the specimen continuously decreases, indicating a transition from ductility to brittleness. This is related to the glassy brittleness of the specimen after melting and solidification at 1000–1200 °C. It is worth noting that the peak strain increased by 81.8% between 400 and 600 °C. Furthermore, from the curve of peak strain variation with temperature, it can be observed that the growth rate slows down from 600 °C onwards. Therefore, 600 °C can be used as the threshold temperature for the increase in peak strain.

**Figure 16:**
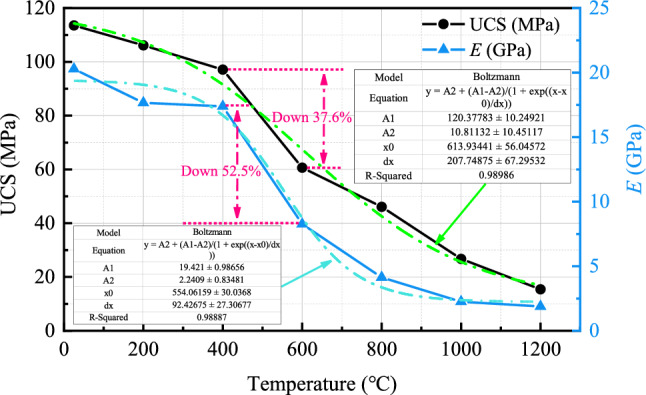
The variation of UCS and elastic modulus with temperature.

The elastic modulus (*E*) and UCS are two important parameters for the stability analysis of rock mass engineering structures. The elastic modulus refers to the tangent modulus, which is determined by the linear elastic part of the stress–strain curve under investigation. The variations of elastic modulus and UCS of granite specimens with temperature are shown in Fig. 16. As the temperature continuously increases, the linear elastic phase of the stress–strain curve gradually becomes flat, leading to a decrease in elastic modulus. The elastic modulus is a physical quantity that describes the material’s resistance to deformation, and a decrease in elastic modulus signifies a decrease in the ability of granite specimens to resist deformation. The fundamental reason for the continuous decrease in elastic modulus of thermally damaged granite specimens mentioned in this article is not only the mineral decomposition and crystal structure damage caused by high temperatures but also the destruction of the originally compacted cementation structure between mineral particles due to high temperatures, resulting in a looser distribution of mineral grains. Consequently, the frictional force used to resist compressive deformation within the specimens decreases sharply. Similarly, UCS also gradually decreases with increasing temperature. The maximum reductions in elastic modulus and UCS occur at temperatures between 400 and 600 °C, with reductions of 52.50% and 37.56%, respectively. Furthermore, from the temperature variation curves of the elastic modulus and UCS, it can be observed that both their slopes begin to decrease to varying degrees at 600 °C, indicating a slowing down of the loss of elastic modulus and UCS. This not only corresponds to the threshold temperature for longitudinal wave velocity loss of thermally damaged granite specimens in this study but also corresponds to the threshold temperature for peak strain growth. Therefore, 600 °C can be used as the thermal damage threshold for granite in this research.

## Conclusions

This article analyzes the physical and mechanical behavior of thermally damaged granite from macroscopic and microscopic perspectives using methods such as ultrasonic testing, polarized light microscopy observation, X-ray diffraction, X-ray fluorescence spectroscopy analysis, and mechanical experiments. It elucidates the thermal damage mechanism of granite from the angle of mineral composition and microstructure evolution with temperature. The main conclusions are as follows:

1. Changes in the physical properties of specimens exhibit three characteristic temperature points at 400 °C, 800 °C, and 1000 °C. Below 400 °C, there is no significant change in the mass, volume, and density of the specimens. When the heating temperature rises from 400 to 800 °C, the mass loss rate of the specimens increases from 0.219 to 0.499%, the volume expansion rate increases from 0.681 to 6.10%, and the density loss rate increases from 0.894 to 6.22%. When the heating temperature rises from 1000 to 1200 °C, the mass loss rate of the specimens increases from 0.318 to 0.357%, the volume expansion rate increases from 6.06 to 7.96%, and the density loss rate increases from 6.01 to 7.71%.
2. The longitudinal wave velocity of specimens decreases continuously with increasing temperature. At 1200 °C, an abnormal rebound in wave velocity occurs due to the specimens solidifying into a glassy phase after melting. The threshold temperature for longitudinal wave velocity loss is 600 °C.
3. With the aggravation of thermal damage, grain boundaries evolution forms intergranular cracks, intragranular cracks evolution forms transgranular cracks, which further develop into large, structurally complex transgranular cracks, and finally, these cracks interpenetrate and spread to form a penetrating crack network. Minerals with natural cleavage structures, such as feldspar and biotite, exhibit significantly more cracks than quartz.
4. The temperature has little effect on the contents of major elements in granite, but it alters the mineral composition and microstructural characteristics of granite, thereby influencing its mechanical properties. Quartz diffraction intensity begins to significantly increase at 400 °C, while at 1200 °C, the diffraction intensity of quartz decreases significantly and quartz starts to decompose. The overall trend of diffraction intensity for biotite and feldspar minerals is decreasing. albite rapidly decomposes at 800 °C, and at 1000 °C, potash feldspar disappears, resulting in the formation of anorthoclase with poorer structural properties, thus altering the mechanical properties of the granite. The FWHM parameter values of the major minerals in granite decrease to varying degrees with increasing temperature, while the grain volume of minerals increases. This leads to the destruction of the cementation structure inside the specimen and promotes the development of microcracks.
5. The stress–strain curve indicates that the granite specimen has undergone two opposite processes of transition, from brittle to ductile and from ductile to brittle, which corresponds to the trend of the peak strain increasing initially and then decreasing with temperature. The elastic modulus and UCS gradually decrease with increasing temperature. 600 °C is not only the threshold temperature for an increase in peak strain but also the threshold temperature for a decrease in elastic modulus and UCS. Therefore, 600 °C serves as the threshold for thermal damage in this study.

### Supplementary Information

Supplementary Information.

## Data Availability

All data generated or analysed during this study are included in this published article [and its Supplementary information files].
